# Supplementary material for: Influence of N-Glycosylation on Virus–Host Interactions in Halorubrum lacusprofundi
Source: Viruses. 2023 Jun 28;15(7):1469. doi: 10.3390/v15071469 (PMC10384203; doi:10.3390/v15071469)
Supplement: Supplementary file 1 [file viruses-15-01469-s001.zip › viruses-2401318-supplementary.pdf]

Supplementary Material

Table of contents

Supplementary Figures

Supplementary Figure S1: Glycoprotein staining of cells and infectious agents.

Supplementary Figure S2: The Δ*aglB* mutant shows decreased adaptability to changes in sodium chloride concentrations, as compared to the parent strain.

Supplementary Figure S3: Viral and plasmid genome copy numbers in cell pellets for the three infectious agents.

Supplementary Tables

Supplementary Table S1: Primers (PCR and qPCR) used in this study.

Supplementary Figures

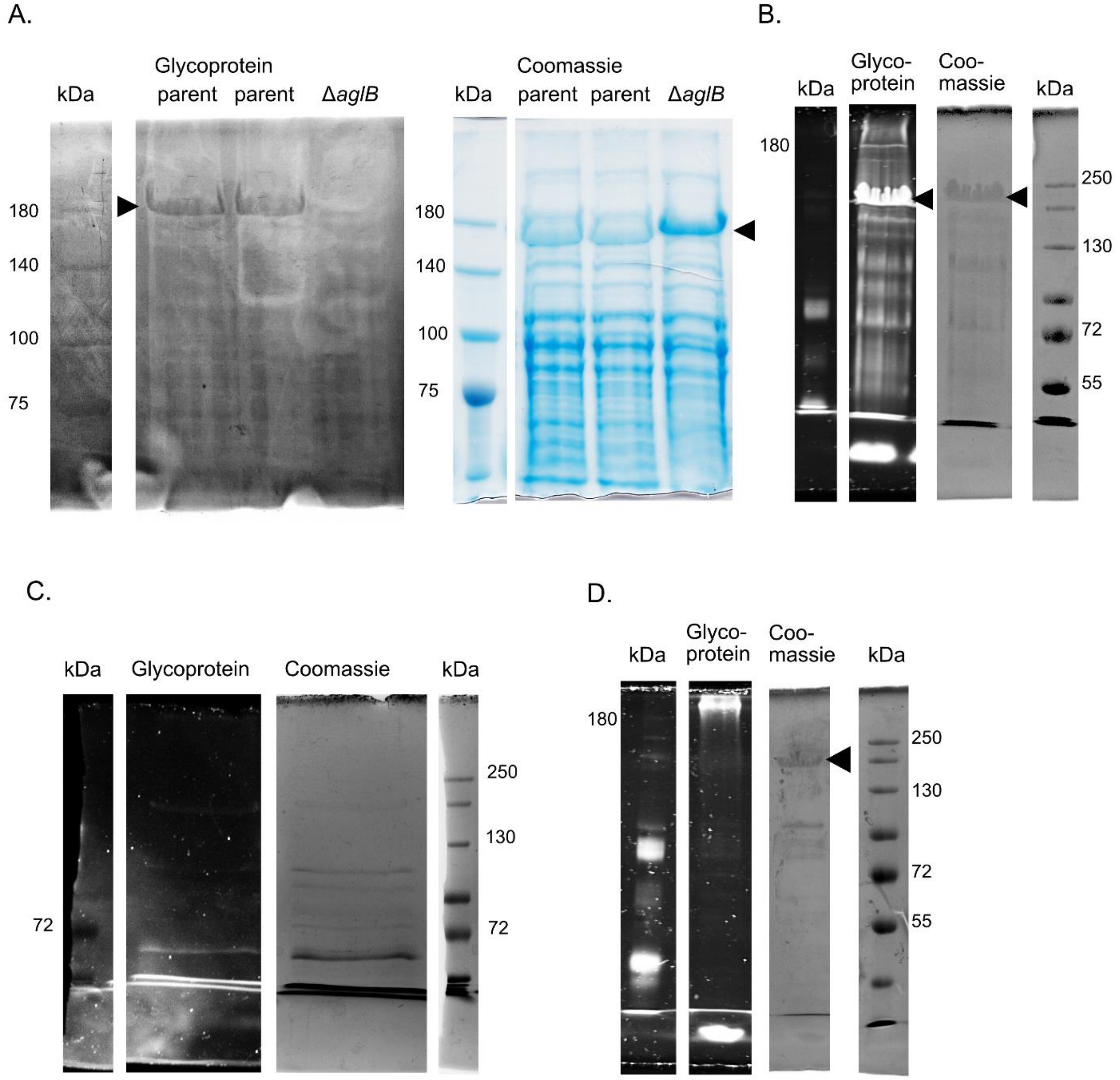

Supplementary Figure S1: Glycoprotein staining of cells and infectious agents.
Cell preparations from parent and Δ*aglB* strains **(A)** were separated via SDS-PAGE (8% Acrylamide) and stained with either periodic acid-Schiff staining or Coomassie dye to visualize all proteins. ExcelBand 3-color Regular Range Protein Marker (Bio Lab) was added a as a size standard (kDa), the position of the S-layer glycoprotein is marked with black arrows in this and all following graphs. Purified preparations of HFPV-1 **(B),** HRTV-DL1 **(C)** and PVs **(D),** produced in their native host organisms, were separated via SDS-PAGE (8% Acrylamide). Glycoproteins were visualized with the Pro-Q Emerald 300 Glycoprotein Kit (Invitrogen) after which gels were stained with Coomassie Dye to visualize all proteins. CandyCane Glycoprotein Standard (Invitrogen, left) and broad Range Color Prestained Protein Standard (NEB, right) were added as size markers (kDa).

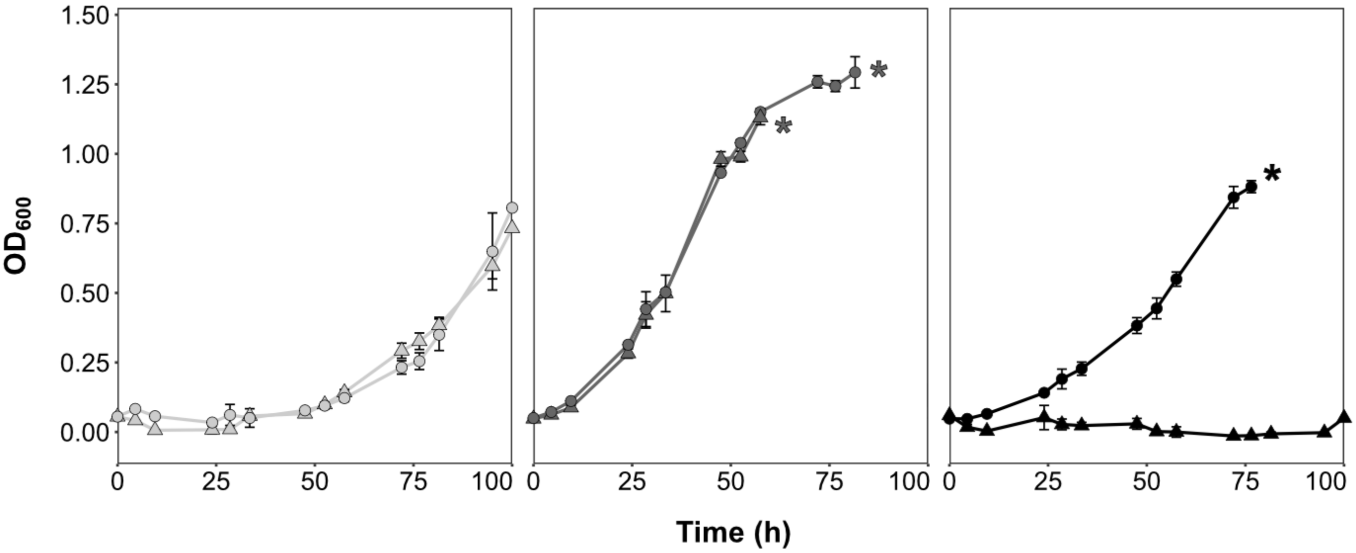

Supplementary Figure S2: The Δ*aglB* mutant shows decreased adaptability to changes in sodium chloride concentrations, as compared to the parent strain.

The growth of the parent (Δ*pyrE2,* circles) and Δ*aglB* (triangles) strains was monitored in media containing NaCl concentrations of 100 g/l = 1.7 M (light grey), 180 g/l = 3.1 M (dark grey) or 250 g/l = 4.3 M (black). The growth data for 180 g/l was generated in a separate experiment to the data shown in Figure 1C, simultaneously with the 100 g/l and 250 g/l cultures. Each point represents the average of three biological replicates ± standard deviation of the mean. Asterisks indicate when cultures went into biofilm and OD_{600} could no longer be accurately measured.

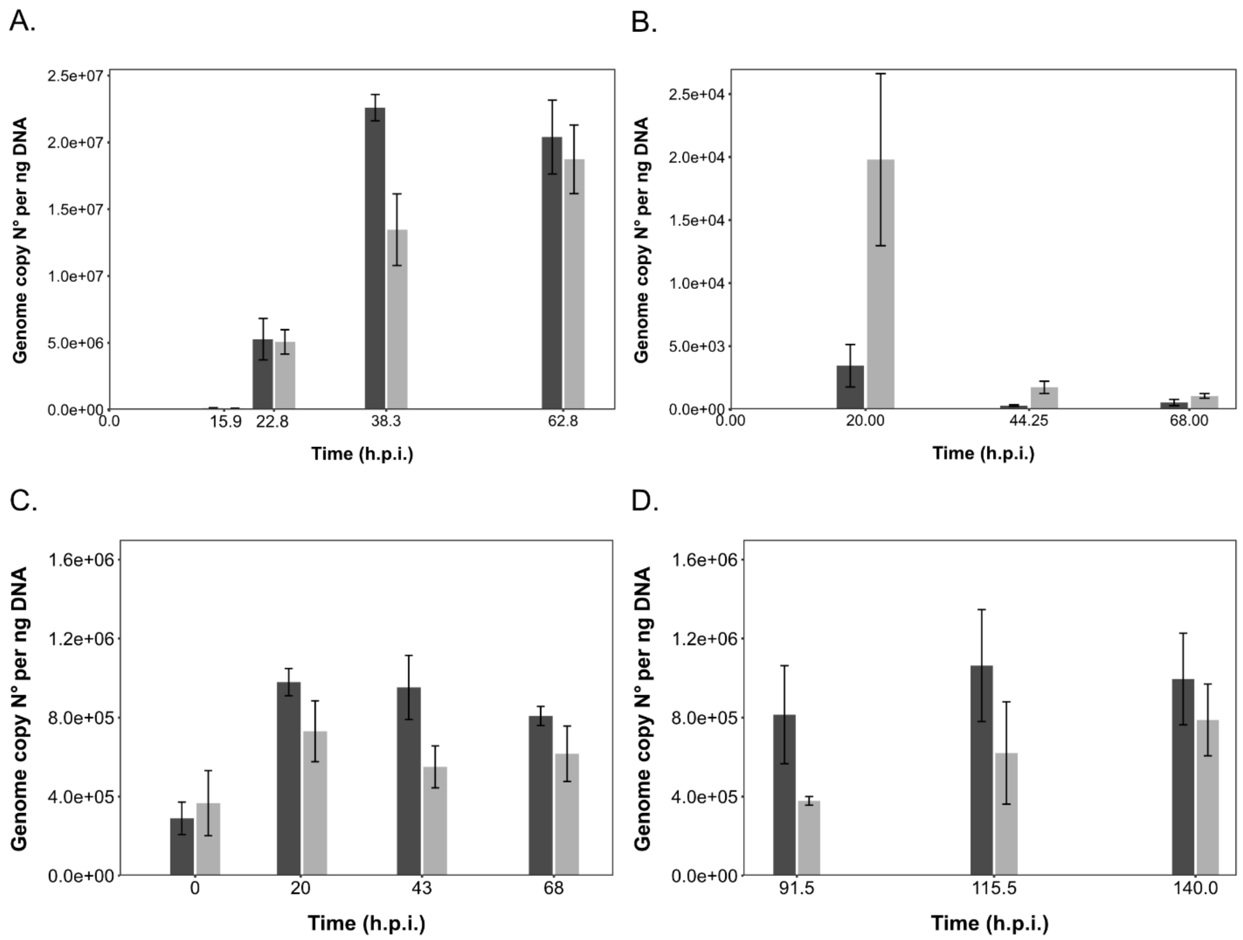

Supplementary Figure S3: Viral and plasmid genome copy numbers in cell pellets for the three infectious agents.

Viral or plasmid gcns per ng of DNA within cells upon infection with HRTV-DL1 **(A)** (Figure 3), HFPV-1 **(B)** (Figure 4), initial **(C)** and extended infection **(D)** with plasmid pR1SE (Figure 5). Bars represent the average of three biological replicates ± standard deviation of the mean for the parental strain (dark grey) and the Δ*aglB* mutant (light grey).

Supplementary Tables

Supplementary Table S1: Primers (PCR and qPCR) used in this study. Nucleotides in lowercase correspond to the exon-flanking regions of the *Hlac_1062* (*aglB*) gene.

Name	Target	Sequence 5' -> 3'	qPCR conditions: Annealing temperature, primer concentration
Hlac_1062-up FW	*Hrr. lacusprofundi Hlac_1062* (*aglB*) upstream fragment, including *Eco*RI restriction site (forward)	CTAGTGGATCCCCCGGGCTGCAGGAATTCcgaatccgcgatgcgtcacg	Not applicable
Hlac_1062-up REV	*Hrr. lacusprofundi Hlac_1062* (*aglB*) upstream fragment (reverse)	gatcaggcggcgggcgggaTCAGGCGCTCAttacgtgaagacgactgtc	Not applicable
Hlac_1062-down FW	*Hrr. lacusprofundi Hlac_1062* (*aglB*) downstream fragment (forward)	gttttgacagtcgtcttcacgtaATGAGCGCCTGatcccgcccgcc	Not applicable
Hlac_1062-down REV	*Hrr. lacusprofundi Hlac_1062* (*aglB*) downstream fragment, including *Hind*III restriction site (reverse)	CCCCTCGAGGTCGACGGTATCGATAAGCTTggaccggcggcgagctcgaaag	Not applicable
Hlac_1062 KO FW	PCR screening primer for *Hrr. lacusprofundi Hlac_1062* (forward)	CGAATCCGCGATGCGTCACG	Not applicable
Hlac_1062-2 FW	PCR screening primer for *Hrr. lacusprofundi Hlac_1062* (forward)	CGTTCCGACGACGCGAACC	Not applicable
Hlac_1062 KO REV	PCR screening primer for *Hrr. lacusprofundi Hlac_1062* (reverse)	GAGCTCGAAAGCGCCGTCG	Not applicable

16S rRNA FW	*Hrr. lacusprofundi* 16S rRNA for quantification (forward)	CGTGGCGAATAGCTCAGTAA	
16S rRNA REV	*Hrr. lacusprofundi* 16S rRNA for quantification (reverse)	TTCCAGGTGGATTGTGGTATG	
Hlac_1062 FW	*Hrr. lacusprofundi Hlac_1062* for quantification (forward)	CATCATGGAGAACTACCCGAATC	
Hlac_1062 REV	*Hrr. lacusprofundi Hlac_1062* for quantification (reverse)	CATGATGTGGTCCCAGAGTG	
TyrVUF	HFPV-1 infection detection	ACGAACGAGAACACCGACC	Not applicable
TyrVUR	HFPV-1 infection detection	TGATGACGAATCCAACGAGCAG	Not applicable
FVP3	VP3 of HFPV-1 for quantification (forward)	TTGCGTACGCGGTATCTGTC	68 °C, 0.13 µM
RVP3	VP3 of HFPV-1 for quantification (reverse)	AGCTTCTCCGCATCGTCTTT	68 °C, 0.13 µM
qPMC2 F	CH1 of all *Hrr. lacusprofundi* strains for quantification (forward)	GAGTTAGTGAAGTATCTTCG	61 °C, 0.15 µM
qPMC2 R	CH1 of all *Hrr. lacusprofundi* strains for quantification (reverse)	GCTCTACATCCTCATAATAC	61 °C, 0.15 µM
HRTV-DLF	HRTV-DL1 genome test for infection (forward)	CTAACAGCACGCCAAGAGGA	Not applicable
HRTV-DLR	HRTV-DL1 genome test for infection (reverse)	CACCACTGGTTTGCTTTCCG	Not applicable
BV37VPF	HRTV-DL1 genome for quantification (forward)	CACGCTCTCGGAAGCAAACC	71 °C, 0.2 µM
BV37VPR	HRTV-DL1 genome for quantification (reverse)	CTCGGAGTCGCCATACTGGG	71 °C, 0.2 µM

ORF6_seq1F	ORF6 of pR1SE to test for infection (forward)	CAATCATTTTCTGATTCGGAAGC	Not applicable
ORF6_seq1R	ORF6 of pR1SE to test for infection (reverse)	GAGGGTTGTGAGTCGTTGTAG	Not applicable
qORF6F	ORF6 of pR1SE for quantification (forward)	ATCGACGACGCAGCCAACAC	67.8 °C,0.125 µM
qORF6R	ORF6 of pR1SE for quantification (reverse)	GTGATTGCGTCCGGGTTGAG	67.8 °C,0.125 µM
